# Microsurgical Anatomy of the Jugular Foramen Applied to Surgery of Glomus Jugulare via Craniocervical Approach

**DOI:** 10.3389/fsurg.2020.00027

**Published:** 2020-05-15

**Authors:** Felipe Constanzo, Mauricio Coelho Neto, Gustavo Fabiano Nogueira, Ricardo Ramina

**Affiliations:** ^1^Department of Neurological Surgery, Clinica Bio Bio, Concepcion, Chile; ^2^Neurosurgery Department, Neurological Institute of Curitiba, Curitiba, Brazil; ^3^Otolaryngology Department, Neurological Institute of Curitiba, Curitiba, Brazil

**Keywords:** jugular foramen, skull base, surgical anatomy, paragangliomas, craniocervical approach

## Abstract

The jugular foramen remains one of the most complex regions of the human body. Approaching lesions in this area requires extensive anatomical knowledge and experience, due to the many critical neurovascular structures passing through or around the jugular foramen. Here, we present a concise review of the microsurgical anatomy of the jugular foramen in relation to the craniocervical approach.

## Introduction

Since the beginning of modern neurosurgery, tumors of the jugular foramen have been regarded as inaccessible due to the complex bony and neurovascular anatomy, and significant deficits associated with attempted resection (1). Nonetheless, several otologists and neurosurgeons from the early twentieth century have described different approaches to this region, with varying success (2), and have further contributed to the anatomical basis for modern approaches. The objective of this article is to present this complex anatomy as simply as possible, and to apply this anatomy to surgically approach the jugular foramen.

## General Features

**Figure 1:**
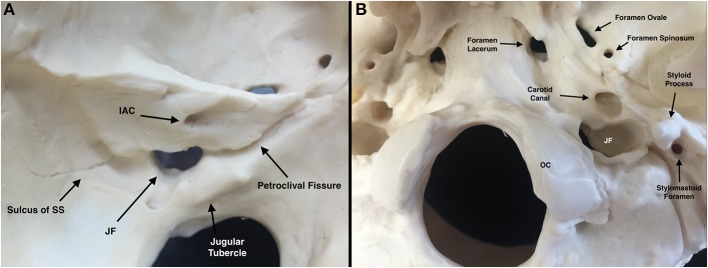
Osseous jugular foramen (JF) and related structures [Modified from Ramina and Tatagiba (3)]. **(A)** Left intracranial view of JF. **(B)** Left extracranial view of JF. IAC, internal auditory canal; JF, jugular foramen; OC, occipital condyle; SS, sigmoid sinus.

The jugular foramen is a transition zone between structures of the ear, posterior fossa and cervical region, and exposition of one or more of these areas may be necessary to completely resect lesions growing from, or extending into the jugular foramen. Its location in the medial-inferior surface of the petrous pyramid—in the transition of the occipital and temporal bones—implies that besides the structures that transverse the foramen, the carotid artery, internal auditory canal, labyrinth, facial nerve, hypoglossal nerve and brainstem, may be compromised or be exposed during surgery [Figure 1; (4, 5)].

**Figure 2:**
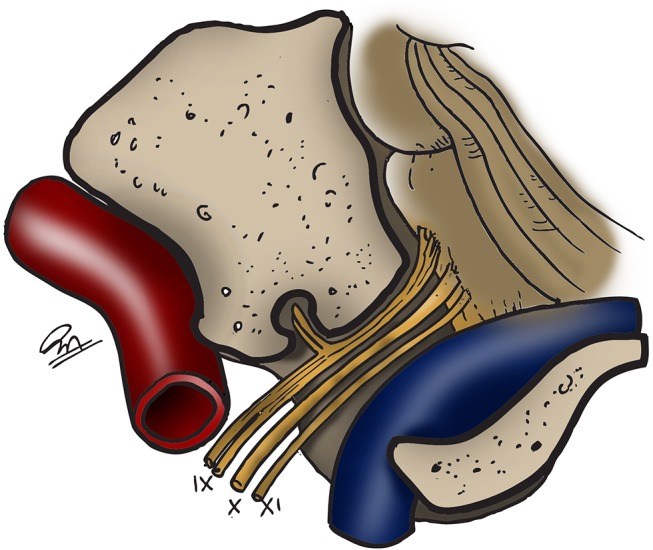
Schematic view of the structures of the jugular foramen. Carotid artery in red, sigmoid sinus and jugular bulb in blue (3). IX: glossopharyngeal nerve. X, vagus nerve. XI, accessory nerve. [Modified from Ramina and Tatagiba (3)].

**Figure 3:**
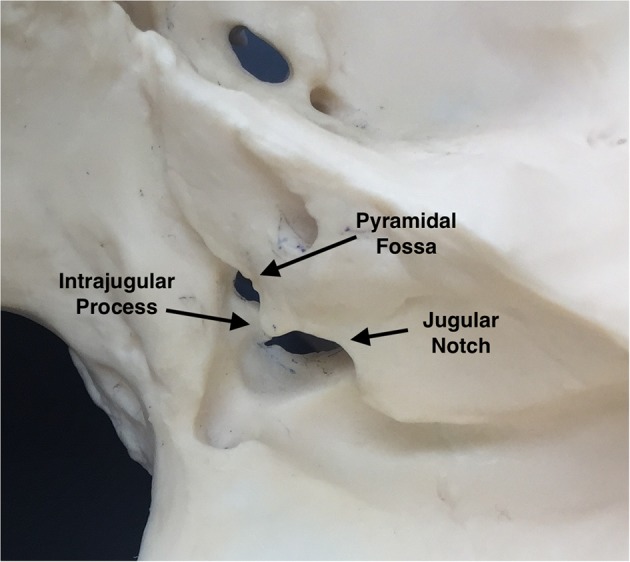
Parts of the roof of the jugular foramen (right intracranial view) [Modified from Ramina and Tatagiba (3)].

The jugular foramen is usually divided in 2 portions (6): The *pars nervosa*, housing the glossopharyngeal nerve and its tympanic branch (Jacobson's nerve), the inferior petrosal sinus and the meningeal branches of the ascending pharyngeal artery; and the *pars vascularis*, containing the sigmoid sinus, vagus (and its auricular branch) and accessory nerves (Figure 2). The *pars nervosa* is located anteromedial to the *pars vascularis*, and they are separated by a dural septum (7). On its intracranial surface, the roof of the jugular foramen is divided into the pyramidal fossa, intrajugular process, and jugular notch, covering the pars nervosa, dural septum, and pars vascularis, respectively, serving as reference points for suprajugular approaches (8) (Figure 3). The deep end of the pyramidal fossa lodges the opening of the canaliculus cochleae.

On its extracranial surface, the jugular foramen has an oblique course from medial to lateral and from posterior to anterior, and it is located posterior to the carotid canal, anterolateral to the occipital condyle, and medial to the styloid process (Figure 1B).

## Vascular Anatomy

**Figure 4:**
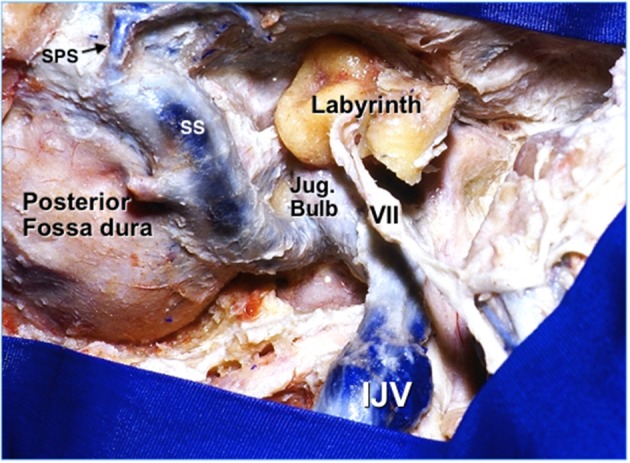
Lateral extradural exposure of a right jugular foramen (3). IJV, internal jugular vein. Jug, jugular. SPS, superior petrosal sinus. SS, sigmoid sinus. VII, facial nerve. [Modified from Ramina and Tatagiba (3)].

Most of the jugular foramen is occupied by the jugular bulb, formed by the union of the sigmoid sinus and the inferior petrosal sinus, and is continued as the internal jugular vein in the cervical compartment (Figure 4). The jugular bulb has a size of approximately 15 mm wide and 20 mm high (9), though there is a notorious asymmetry of both sides, with the right side being wider in most patients (10). Larger jugular bulbs may erode through the middle ear or internal auditory canal, which have important implications for surgery of vestibular schwannoma and other lesions of the internal auditory canal.

**Figure 5:**
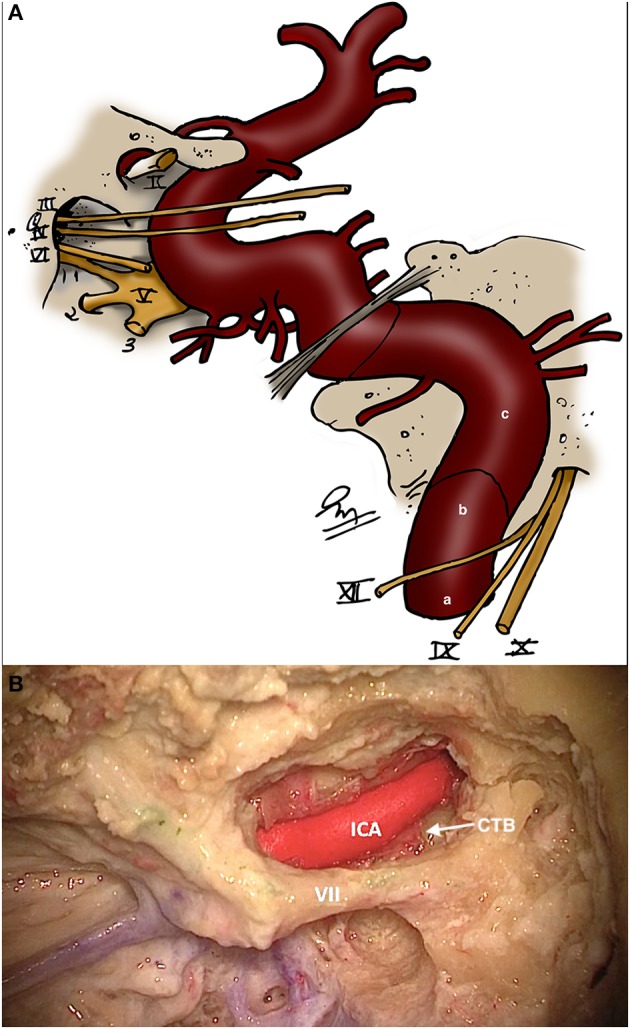
**(A)** Schematic showing the three portions of the petrous segment of the internal carotid artery [Modified from Ramina and Tatagiba (3)]. (a) Ascending (vertical). (b) Genu. (c) Horizontal. **(B)** Horizontal segment of the carotid artery in the petrous bone and its relationship with the facial nerve and caroticotympanic branches (CTB). III, oculomotor nerve; IV, trochlear nerve; V, trigeminal nerve; VI, abducens nerve; VII, facial nerve; IX, glossopharyngeal nerve; X, vagus nerve; XI, accessory nerve.

**Figure 6:**
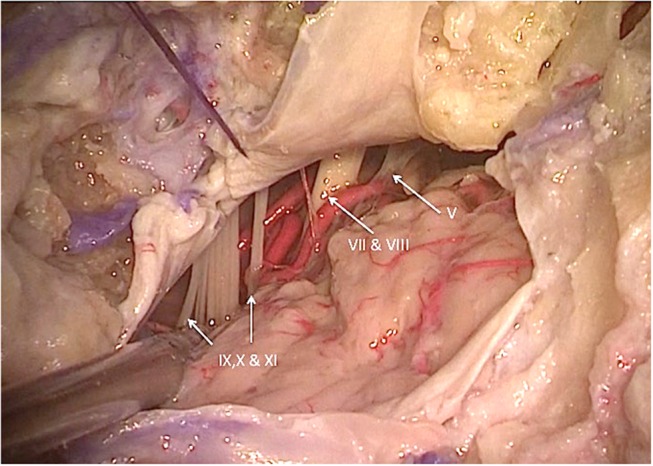
Left lateral cerebellopontine angle view showing the intradural relationships of the nerves of the jugular foramen, internal auditory canal, and vertebral artery and its branches (3). V, trigeminal nerve; VII, facial nerve; VIII, vestibulocochlear nerve; IX, glossopharyngeal nerve; X, vagus nerve; XI, accessory nerve. [Modified from Ramina and Tatagiba (3)].

Branches of the ascending pharyngeal and occipital arteries also pass through the jugular foramen, and provide most of the arterial supply of paragangliomas and meningiomas of this area, thus, being suitable for preoperative embolization (11). The internal carotid artery lays in close relationship with the jugular foramen. It enters the skull through the carotid canal, located anteromedial to the jugular foramen, and inside the petrous bone, three segments are identified: ascending (vertical), genu and horizontal portion (Figure 5), passing anterior to the tympanic cavity, eustachian tube and labyrinth. Caroticotympanic branches of the internal carotid artery emerge from this segment and may feed the middle ear extension of a jugular tumor (Figure 5B). Several variations of the carotid artery within the temporal bone, such as a dehiscent carotid canal or aberrant carotid artery, may produce an unintentional exposition or even lesion of it during approaches to the jugular foramen, and must always be evaluated. It is important to remember that these anomalies, as well as a high-riding jugular bulb may be mistaken for paragangliomas. Finally, the intradural vertebral artery is intimately related to the accessory and vagus nerve in the cerebellopontine angle cistern (Figure 6), and its extradural segment is routinely exposed (and sometimes transposed) in approaches to the jugular foramen.

## Nervous Anatomy

Intradurally, the glossopharyngeal, vagus, and accessory nerves leave the intracranial compartment through the jugular foramen. These nerves leave the medulla and upper cervical spinal cord and transverse the lateral cerebellomedullary cistern before entering the jugular foramen (Figure 6). The glossopharyngeal nerve is usually formed by a single fascicle, whereas the vagal nerve is usually formed by multiple fascicles, and the accessory nerve is formed by multiple rootlets that coalesce into two fascicles, one spinal and one cranial before entering the foramen (12).

Inside the jugular foramen, the glossopharyngeal nerve enters anterior, medial and superior to the X and XI cranial nerves, at the level of the pyramidal fossa, and the vagus and accessory nerves enter at the level of the intrajugular process. All of the nerves are medial to the jugular bulb inside the jugular foramen, which is an important anatomical parameter because it allows a posterior surgical approach to the jugular bulb with preservation of the nerves. Inside the foramen, these nerves cross a connective tissue septum that is in continuity with the pericranium and dura mater (7). The tympanic branch of the glossopharyngeal nerve (Jacobson's nerve) branches off at the level of the inferior ganglion, entering the inferior tympanic canaliculus to form the tympanic plexus to form the lesser petrosal nerve in the middle fossa. The auricular branch of the vagus nerve (Arnold's nerve) is also formed at the level of the jugular foramen, from the superior ganglion of the vagus and inferior ganglion of the glossopharyngeal nerves, passing through the mastoid canaliculus in direction to the external auditory canal, crossing the mastoid segment of the facial nerve (and giving off a small branch).

**Figure 7:**
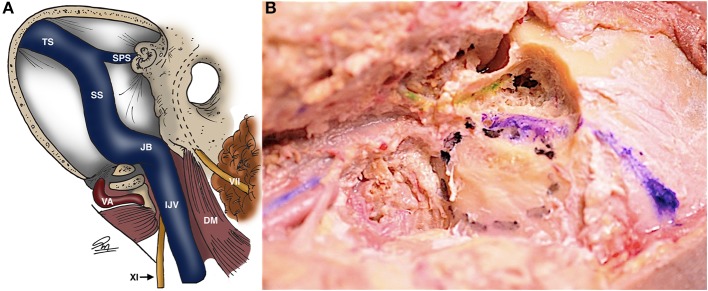
Schematic **(A)** and cadaveric **(B)** views of the jugular foramen with its vascular relationships and the facial nerve in the fallopian canal [dashed lines in **(A)**, green in **(B)**]. Transverse and sigmoid sinuses are blue in **(B)** [Modified from Ramina and Tatagiba (3)]. DM, digastric muscle; IJV, internal jugular vein; JB, jugular bulb; SPS, superior petrosal sinus; SS, sigmoid sinus; TS, transverse sinus; VA, vertebral artery. VII, facial nerve; XI, glossopharyngeal nerve.

**Figure 8:**
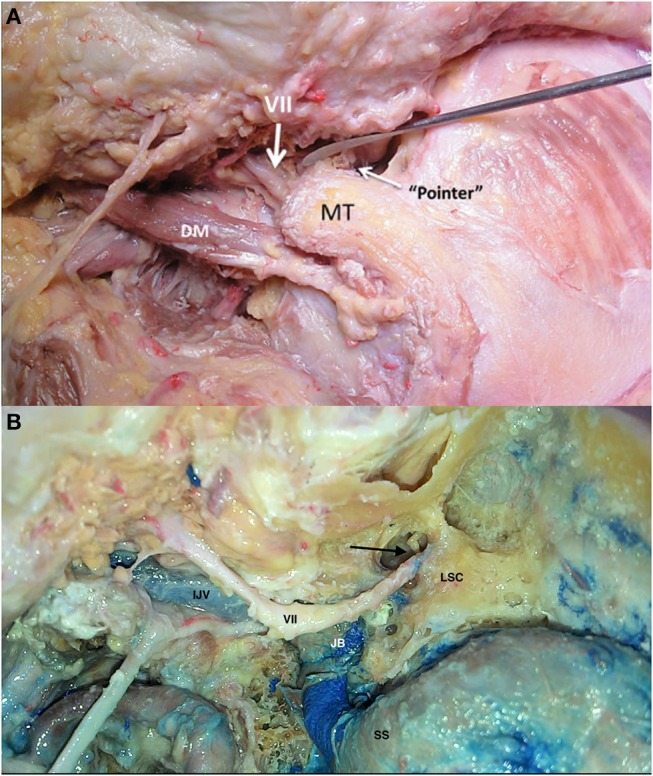
Landmarks to identify the facial nerve [Modified from Ramina and Tatagiba (3)]. **(A)** Extracranial facial nerve location in relation to the tragal pointer, mastoid tip (MT) and digastric muscle (DM). **(B)** Facial nerve after mastoidectomy, with the incus pointing toward the transition between the tympanic and mastoid segment of the facial nerve (arrow). IJV, internal jugular vein; JB, jugular bulb; LSC, lateral semicircular canal; SS, sigmoid sinus; VII, facial nerve.

Even though not a part of the jugular foramen, the facial and vestibulocochlear nerves are usually exposed during surgery of this region, or they may be compromised by tumor itself. The VII-VIII complex is located superior and medial to the IX-X-XI nerves in the cerebellopontine angle cistern (Figure 6). Inside the temporal bone, the labyrinth becomes an important landmark of the superomedial margin of the mastoidectomy (Figure 4), whereas the facial nerve in its tympanic and mastoid segments should be protected in lateral approaches, in some cases needing rerouting of the nerve to properly expose the jugular foramen (Figure 7). The anatomical parameters to identify the nerve within the temporal bone are the mastoid tip, the cartilage of the external ear canal (“pointer”) and the posterior belly of the digastric muscle (Figure 8) (13). In cases where a radical mastoidectomy is needed, the mastoid segment of the facial nerve, running anterior and medial to the sigmoid sinus and digastric ridge, is identified after opening the mastoid antrum, where the short process of the incus points to the fallopian canal medial to the lateral semicircular canal (Figure 8B).

Finally, the hypoglossal nerve lays inferior and lateral to the IX-X-XI complex, and the hypoglossal canal is separated from the jugular foramen by the jugular tubercle, where is usually not exposed during surgery, however, in the cervical region the nerve has intimate relationship with the nerves of the jugular foramen.

## Cervical Anatomy

**Figure 9:**
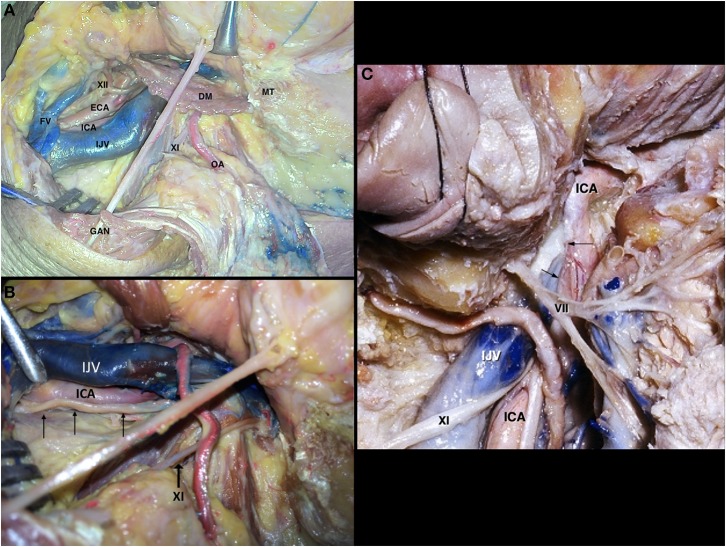
Neck dissection [Modified from Ramina and Tatagiba (3)]. **(A)** Exposition of the skull base and vascular structures. **(B)** Course of the vagus nerve in the proximal cervical region (arrows). **(C)** Relationship of the carotid artery and venous system on the lateral skull base. Arrows represent the space between both structures where the glossopharyngeal, vagus, and accessory nerves exit the skull base. DM, digastric muscle; ECA, external carotid artery; FV, facial vein; GAN, great auricular nerve; ICA, internal carotid artery; IJV, internal jugular vein; MT, mastoid tip; OA, occipital artery; VII, facial nerve; XI, accessory nerve; XII, hypoglossal nerve.

Neck dissection is often performed due to the extension of tumors through the jugular foramen. The surgical anatomy of the neck region related to the jugular foramen is complex and includes different muscles, vessels, and nerves. The muscles are: sternocleidomastoid (SCM), digastric, splenius capitis, obliquus capitis superior and inferior, rectus capitis posterior major and splenius cervicis. The arteries are: common carotid, external carotid and its branches, internal carotid, and vertebral artery at the craniocervical junction. The veins are: common facial, external and internal jugular veins. The nerves are: great auricular nerve, cranial nerves X,XI and XII and the pericarotid sympathetic trunk (Figure 9).

After leaving the jugular foramen, the glossopharyngeal nerve maintains its medial course, following the anterior border of the carotid artery, and further medializing to form the pharyngeal plexus on the surface of the middle pharyngeal constrictor muscle, so its cervical segment is not readily visible during surgery. The vagus nerve, on its more cranial part, runs lateral to the internal carotid artery (Figure 9B), and then courses between the carotid and internal jugular vein inside the carotid sheath, giving off parasympathetic branches on its course to the thorax and abdomen. The accessory nerve emerges in the neck between the carotid and internal jugular vein, but promptly courses posteriorly in direction to the trapezius muscle (Figure 9). The hypoglossal nerve, after leaving its canal, gives off branches to the inferior ganglion of the vagus nerve and superior sympathetic ganglion, then crosses the vagus nerve and internal carotid artery anteriorly, passing behind the digastric muscle to innervate the muscles of the tongue (Figure 9A).

The common, internal and external carotid arteries run medial to the internal jugular vein. In the carotid triangle of the neck—bounded posteriorly by the sternocleidomastoid muscle and superiorly by the stylohyoideus and digastric muscles—the internal carotid artery is posterolateral to the external carotid artery and the internal jugular vein is lateral to both (Figure 9).

**Figure 10:**
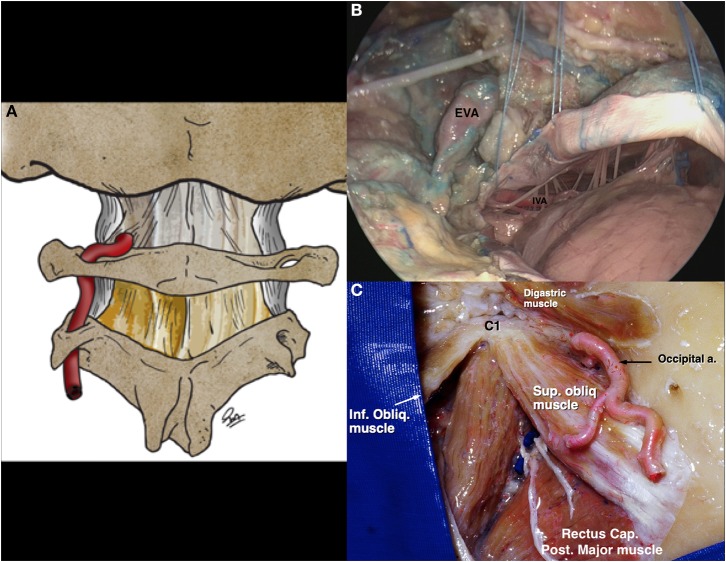
Vertebral artery in the skull base [Modified from Ramina and Tatagiba (3)]. **(A)** Schematic drawing of the course of the cranial vertebral artery before perforating the atlanto-occipital membrane. **(B)** Cadaveric view of the vertebral artery entering the skull, and its relationship with bulbar nerves. **(C)** Suboccipital triangle, limited by the rectus capitis posterior major, superior oblique, and inferior oblique muscles. EVA, extracranial vertebral artery; IVA, intracranial vertebral artery.

The vertebral artery, after leaving the transverse foramen of C2 medial to the rectus capitis lateralis, crosses C2 nerve root to reach the transverse foramen of C1, then coursing medially and behind the occipitoatlantal joint over the vertebral sulcus of C1 to perforate the posterior part of the atlanto-occipital membrane and duramater to enter the cranium (Figures 10A,B). This area of the vertebral artery is bound within the suboccipital triangle, limited by the rectus capitis posterior major, superior oblique, and inferior oblique muscles (Figure 10C). It is worth noting that the vertebral artery is surrounded by a venous plexus that is largest in the region of the C1-C2 joint.

## Craniocervical Approach

**Figure 11:**
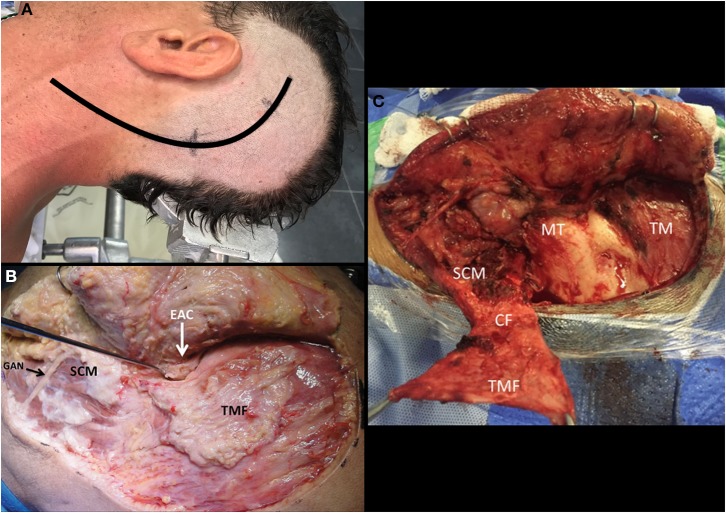
Left-sided craniocervical approach [Modified from Ramina and Tatagiba (3)]. **(A)** Head positioning and incision. **(B)** Suprafascial dissection exposing the temporalis muscle fascia and cervical fascia. **(C)** Creating the temporo-cervical-SCM flap to close the surgical defect after surgery. CF, cervical fascia; EAC, external auditory canal; GAN, great auricular nerve; MT, mastoid tip; SCM, Sternocleidomastoid muscle; TM, temporalis muscle; TMF, temporalis muscle fascia.

**Figure 12:**
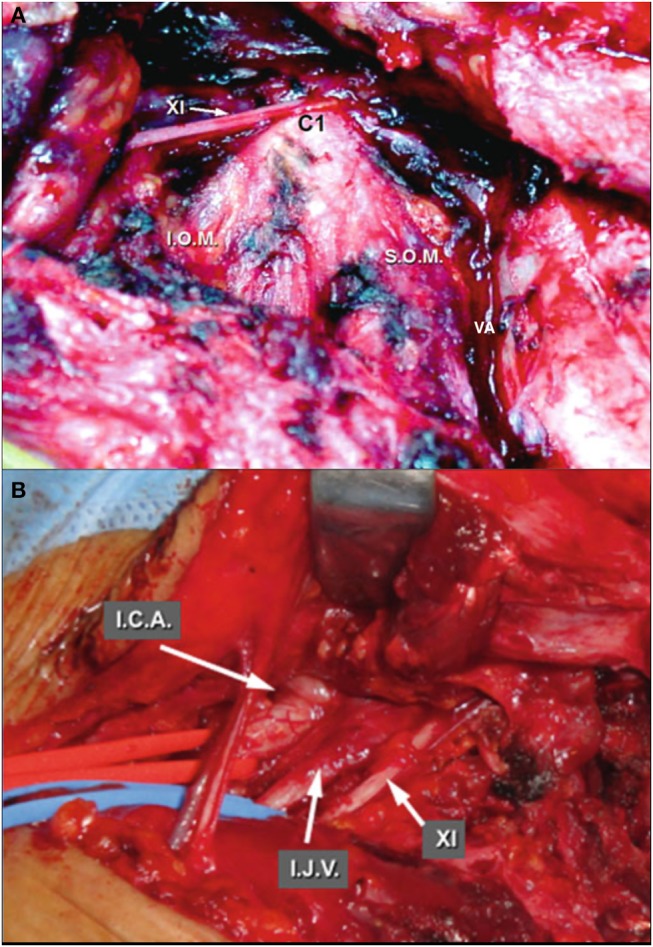
Neck dissection [Modified from Ramina and Tatagiba (3)]. **(A)** Left lateral mass of C1 as landmark to identify the accessory nerve and vertebral artery. **(B)** Deeper dissection, identifying the internal carotid artery, internal jugular vein and accessory nerve. ICA, internal carotid artery; IJV, internal jugular vein; IOM, inferior oblique muscle; SOM, superior oblique muscle; VA, vertebral artery; XI, accessory nerve.

**Figure 13:**
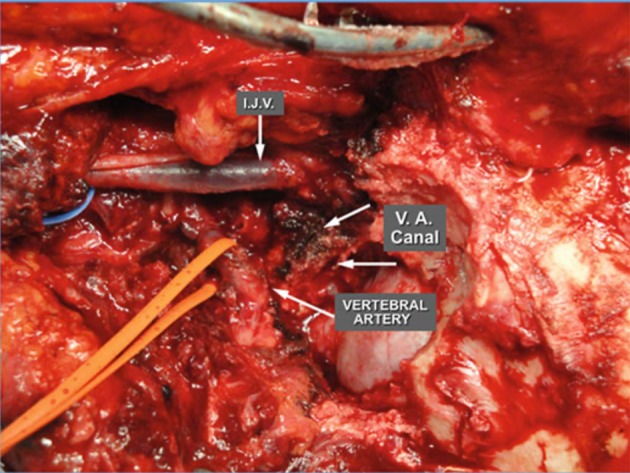
Skull base after neck dissection and transposition of the vertebral artery (3). IJV, internal jugular vein; VA, vertebral artery. [Modified from Ramina and Tatagiba (3)].

**Figure 14:**
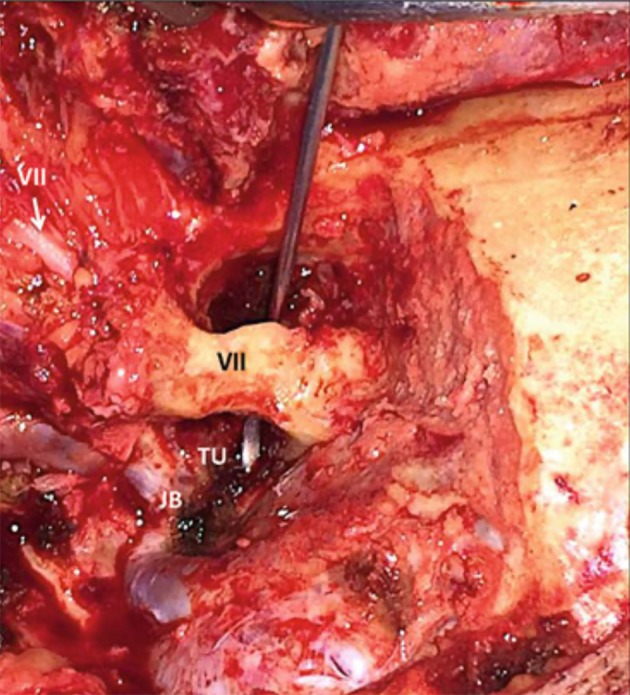
After radical mastoidectomy, the facial nerve is left protected by bone inside the fallopian canal, with a hook passing below the canal to show the working space to resect tumor in the hypotympanum (3). JB, jugular bulb; TU, tumor; VII, facial nerve in the fallopian canal (black) and entering the parotid gland (white).

**Figure 15:**
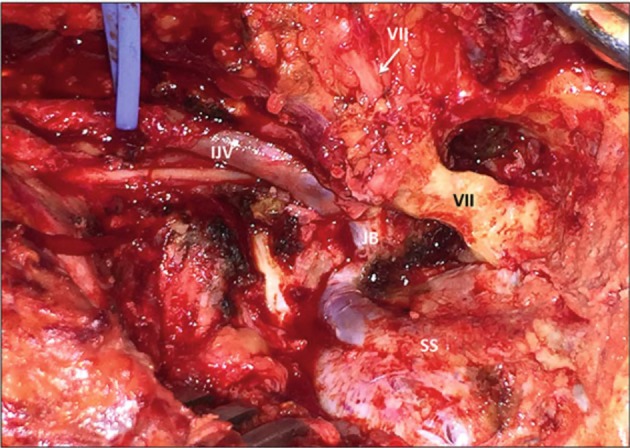
Jugular foramen opened after neck dissection, radical mastoidectomy and retrosigmoid craniectomy (3). IJV, internal jugular vein; JB, jugular bulb; TU, tumor; SS, sigmoid sinus; VII, facial nerve in the fallopian canal (black) and entering the parotid gland (white). [Modified from Ramina and Tatagiba (3)].

A standard craniocervical approach involves neck dissection, mastoidectomy and suboccipital craniotomy, but it is tailored according to the extension of the lesion to be resected. The patient is positioned in dorsal decubitus, with the head turned to the opposite side at a 60–80 degree angle with the ipsilateral shoulder slightly elevated and electrophysiological monitoring of cranial nerves VII, VIII, IX, X, XI, and XII, as well as bilateral somatosensory evoked responses. A C-shaped incision starting 5cm superior to the zygomatic arch in temporal region circumscribing the ear and continuing up to a cervical fold over the medial border of the sternocleidomastoid muscle (SCM) is performed (Figure 11A). A suprafascial dissection is followed anteriorly, dissecting the great auricular nerve and exposing the temporalis muscle fascia, external auditory canal, mastoid tip, and the anterior border of the SCM muscle (Figure 11B). The temporalis muscle fascia is dissected from the muscle, leaving it anchored to the cervical fascia, which is incised close to the external auditory canal and mastoid tip, creating a large flap with base on the SCM, which is then detached from the mastoid (Figure 11C). The external jugular vein and facial vein are ligated and the digastric muscle is identified. The facial nerve is located at the superior border of the digastric muscle and the hypoglossal nerve at its posters-inferior border. The digastric muscle is detached from the mastoid and reflected down to expose the anterior skull base. Neck dissection is then performed, first identifying the transverse process of C1 - which is a landmark to identify the accessory nerve (postero-inferior) and the vertebral artery (postero-superior) (Figure 12A)—and then the carotid artery and internal jugular vein (Figure 12B). This dissection is tailored to expose the inferior pole of the tumor. The superior and inferior capitis obliquus muscles are cut at the insertion in the transverse process of C1 and the

groove of the vertebral artery in the C1 lamina is identified. The vertebral artery may be transposed from its canal in C1 to enlarge the approach to the jugular foramen (Figure 13). The facial nerve then is identified in the stylomastoid foramen by exposing the mastoid tip, the posterior belly of the digastric muscle, the “tragal pointer,” and the tympanomastoid suture (Figure 8A). A radical mastoidectomy and antrotomy are at this point performed to expose the antrum, ossicles, sigmoid sinus, superior petrous sinus, and labyrinth, leaving the facial nerve in the fallopian canal (though some cases may need transposition) (Figure 14). Inside the mastoid, the facial nerve is identified by the lateral semicircular canal and short process of the incus (Figure 8B). The posterior and anterior walls of the external auditory canal are drilled when the tumor extends through the hypotympanum (Figure 14). The internal carotid artery may be exposed by removing the tympanic bone to obtain cranial control of the artery (Figure 5B). A 3cm retrosigmoid craniectomy is then performed to expose the duramater of the posterior fossa, sigmoid sinus, and jugular bulb, opening the jugular foramen (Figure 15). When the jugular bulb is invaded by tumor, the sigmoid sinus and internal jugular vein are ligated and the tumor is resected *en bloc* extradurally. Then, duramater of the posterior fossa is opened and the tumor is resected in the lateral cerebellopontine angle cistern. After total resection, the dura is closed, and the surgical defect is filled using the digastric muscle as graft, followed by a temporalis muscle flap and repositioning of the cervico-temporalis fascia flap in the mastoid cavity, as previously described (14).

## Conclusion

Precise knowledge of the anatomy of the jugular foramen allows skull base surgeons to access the jugular foramen with minimum morbidity and mortality. The complex anatomy of this region demands a dedicated team with experience to tailor the approach to the morphology of the tumor.

## Author Contributions

FC and MC contributed to the conception and design of the manuscript. RR and GN performed the dissections. FC wrote the bulk of the manuscript. All authors revised the manuscript, corrected it, and approved the final version.

## Conflict of Interest

The authors declare that the research was conducted in the absence of any commercial or financial relationships that could be construed as a potential conflict of interest.
